# Molecular Epidemiology of Drug Resistance Genes in Plasmodium falciparum Isolates Imported from Nigeria between 2016 and 2020: Continued Emergence of Fully Resistant *Pfdhfr*-*Pfdhps* Alleles

**DOI:** 10.1128/spectrum.00528-22

**Published:** 2022-09-15

**Authors:** Xiaoxiao Wang, Xuan Zhang, Hualiang Chen, Jiaqi Zhang, Qiaoyi Lu, Wei Ruan, Feng Ling

**Affiliations:** a Zhejiang Provincial Center for Disease Control and Prevention, Zhejiang, People’s Republic of China; Weill Cornell Medicine

**Keywords:** molecular epidemiology, Nigeria, *Plasmodium falciparum*, drug resistance

## Abstract

Malaria poses public health threats worldwide. Nigeria accounted for the highest numbers of cases (26.8%) and deaths (31.9%) among countries where malaria is endemic in 2020. Currently, monitoring molecular markers in imported malaria cases provides an efficient means to screen for emerging drug resistance in countries where malaria is endemic, particularly in those where field surveillance is challenging. Here, we investigated 165 Plasmodium falciparum infections imported from Nigeria to Zhejiang Province, China, between 2016 and 2020. Multiple molecular markers in *k13*, *Pfcrt*, *Pfmdr1*, *Pfdhfr*, and *Pfdhps* were detected. The prevalences and patterns of mutations were analyzed. Polymorphism of *k13* was limited to 5 of 156 (3.21%) isolates. The wild-type CVMNK allele of *Pfcrt* became predominant (65.36%) compared with the triple mutation CVIET. A low frequency (4.73%) of double mutations (N86Y and Y184F) in *Pfmdr1* was observed. The dominant haplotypes of *Pfdhfr* and *Pfdhps* were IRNDI (92.41%) and ISGKAA (36.84%), respectively. The newly discovered mutant I431V was identified in 21.71% of isolates. A “fully resistant” combination of *Pfdhfr-Pfdhps*, IRN-GE, was found in eight (5.67%) samples, which was hardly seen in Nigeria. The current study demonstrated a high frequency of wild-type *Pfcrt*. Limited polymorphism of *Pfmdr1* but a high prevalence of *Pfdhfr* and *Pfdhps* mutations was illustrated. Our data so far serve as comprehensive surveillance of molecular markers of the *k13*, *Pfcrt*, *Pfmdr1*, *Pfdhfr*, and *Pfdhps* genes. Based on our findings, it has become crucial to evaluate the impact of the emerging fully resistant type of *Pfdhfr*-*Pfdhps* as well as its combination with I431V on the efficacy of sulfadoxine-pyrimethamine (SP) in Nigeria.

**IMPORTANCE** Monitoring the current resistance to antimalarial drugs is critical to enable timely action to prevent its spread and limit its impact. The high prevalence of wild-type *Pfcrt* found in our study is an optimistic signal to reevaluate chloroquine (CQ) sensitivity in Nigeria, which is cost-effective and once played a crucial role in the fight against malaria. Based on the continued emergence of fully resistant *Pfdhfr*-*Pfdhps* alleles illustrated in the current investigation, actions are needed in Nigeria, such as national systemic surveillance to monitor their updated epidemiology as well as assessments of their influence on SP efficacy to minimize any public health impact. These findings urge a response to the threat of drug resistance to facilitate appropriate drug policies in the study area.

## INTRODUCTION

Malaria poses public health threats worldwide, especially in sub-Saharan Africa. There were an estimated 241 million cases of malaria in 2020 in 85 countries where malaria is endemic (1). The World Health Organization (WHO) Africa Region accounted for about 95% of cases, namely, 228 million cases (1). Among the countries where malaria is endemic, in 2020, Nigeria accounted for the highest percentages of cases (26.8%) and deaths (31.9%). Compared with 2018, in 2019 in Nigeria, there was an estimated increase of 2.4 million malaria patients, and >60 million cases were reported (2).

As an inexpensive and safe antimalarial drug, chloroquine (CQ) was once the drug most commonly prescribed in Nigeria (3). Sulfadoxine-pyrimethamine (SP), amodiaquine, quinine, halofantrine, and artemether were also used as monotherapies (3). However, the emergence of CQ-resistant parasites was reported in 1987 in the south of Nigeria and then spread throughout the country (4). Nigeria changed its national drug policy from CQ to artemisinin combination therapy (ACT) with artemether-lumefantrine (AL) in 2005 (4, 5). There has been a reduced response of Plasmodium falciparum infection to ACT in six geographical areas of Nigeria, 10 years after ACT was introduced as a first-line treatment (5). Although SP for intermittent preventive treatment has a positive impact on reducing peripheral parasitemia, placental parasitization, and low birth weight, there are also concerns about increasing resistance to SP (6, 7). These observations emphasize the importance of monitoring the current status and investigating the spread of antimalarial drug resistance in Nigeria.

An increased understanding of the molecular mechanisms underlying parasite resistance has led to a more efficient way to observe its trends by tracking the prevalence of molecular markers associated with drug resistance. The WHO updated a list of 10 validated P. falciparum *k13* markers and 11 candidate markers of partial resistance to artemisinin (2). Since the emergence and clonal expansion of R561H mutant parasites were detected in Rwanda in 2014, which shared no genetic relatedness to those previously reported in Southeast Asia, concerns about their spread were raised in African countries (8). The CQ resistance transporter (*Pfcrt*) located on P. falciparum digestive vacuole membranes is associated with CQ treatment failure. Mutant *Pfcrt* (mainly the K76T mutation) is considered to be a highly sensitive and moderately specific marker for CQ treatment failure (9). The P. falciparum multidrug drug resistance gene *Pfmdr1* encodes an ATP-binding cassette drug transporter and is believed to be the only gene associated with multidrug resistance. Mutant *Pfmdr1*, especially N86Y and an increased copy number, influences the sensitivity to a wide range of antimalarials, including CQ, artemisinin derivatives, and amodiaquine (10, 11). Mutations in the dihydrofolate reductase (*Pfdhfr*) and dihydropteroate synthase (*Pfdhps*) genes of P. falciparum are associated with antifolate drug resistance.

In the current study, we collected P. falciparum isolates imported from Nigeria to Zhejiang Province, China, between 2016 and 2020 and analyzed the profiles of the *k13*, *Pfcrt*, *Pfmdr1*, *Pfdhfr*, and *Pfdhps* genes, which are associated with drug resistance, to provide evidence for an update of antimalarial policies.

## RESULTS

### Overview of malaria infections imported from Nigeria to Zhejiang Province from 2016 to 2020.

**FIG 1:**
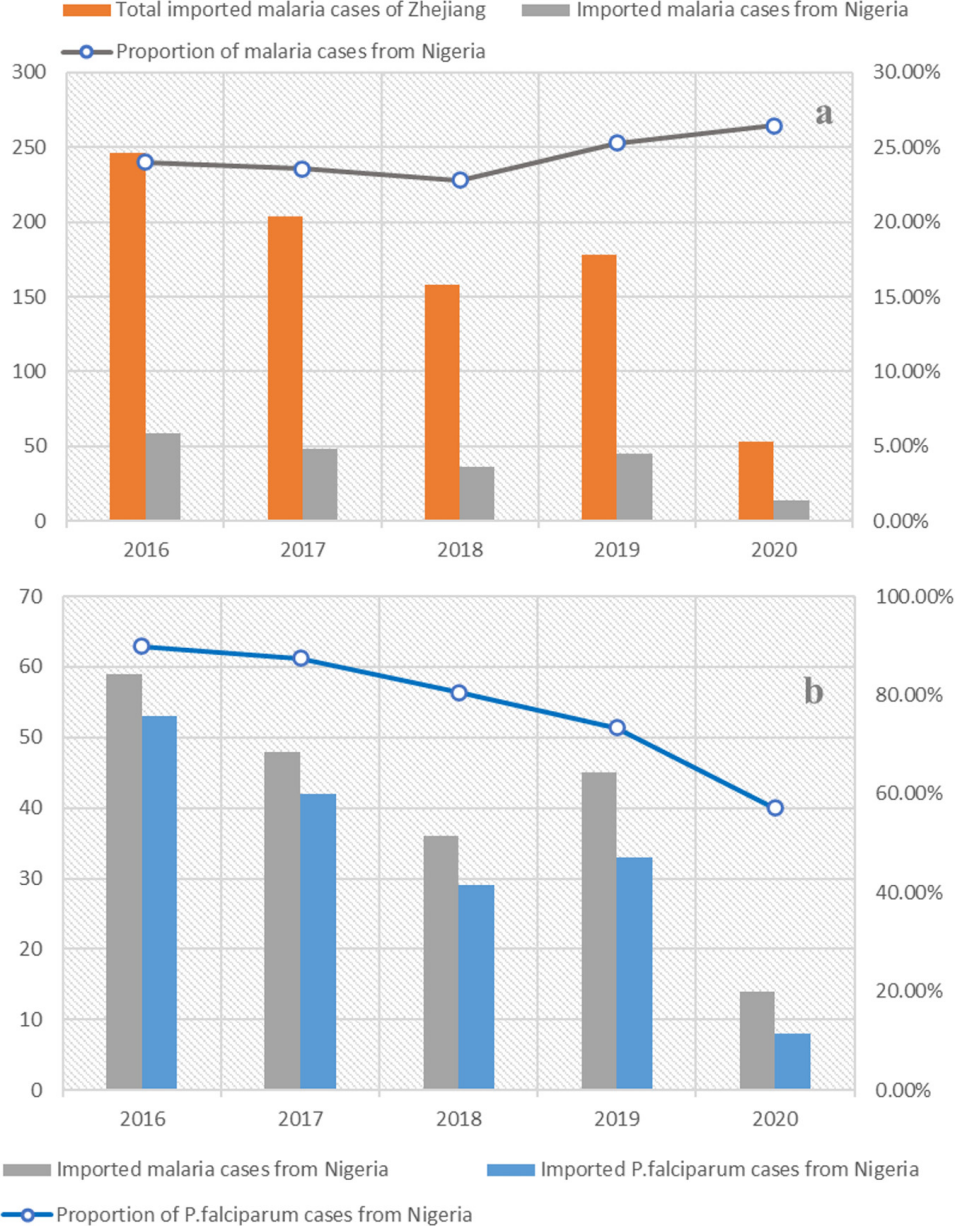
Proportion of imported malaria cases from Nigeria in Zhejiang Province (a) and proportion of P. falciparum cases from Nigeria (b).

A total of 839 malaria cases were imported to Zhejiang Province between 2016 and 2020. Nigeria was the main country of origin, accounting for 24.08% (202/839) of the total (Fig. 1; see also Table S2 in the supplemental material). Among the 202 cases imported from Nigeria, 81.68% (165/204) were caused by P. falciparum, followed by Plasmodium ovale (13.86%; 28/202). The proportions of cases infected by Plasmodium malariae and Plasmodium vivax were 2.97% (6/202) and 1.49% (3/202), respectively. All of the P. falciparum cases imported from Nigeria were included in the current survey. The median (range) age of the study population was 44 (29 to 69) years, and 152 were male. The numbers of P. falciparum samples from 2016 to 2020 were 53, 42, 29, 33, and 9, respectively.

### Prevalence and patterns of *k13* mutations.

A total of 156 sequences of *k13* were successfully obtained from 165 samples. Sequence alignment revealed that 7 (4.49%; 7/156) isolates harbored a nucleotide mutation: C1321T in 2 isolates, A1372G in 1, C1767G in 2, G1948T in 1, and G2026T in 1. The prevalence of amino acid mutations was 3.21% (5/156). Specifically, nucleotide mutations C1321T, A1372G, G1948T, and G2026T resulted in amino acid mutations P441S, N458D, V650F, and A676S, respectively, while nucleotide mutation C1767G did not yield a nonsynonymous amino acid mutation (Table 1).

**TABLE 1:** Prevalence of point mutations in *k13*, *Pfcrt*, *Pfmdr1*, *Pfdhfr*, and *Pfdhps* in P. falciparum isolates imported to Zhejiang Province from Nigeria

Gene	Codon mutation	No. of isolates from yr					Total no. of isolates (%)
		2016	2017	2018	2019	2020	
*k13* (*n* = 156)	P441S	0	1	1	0	0	2 (1.28)
	N458D	0	0	0	1	0	1 (0.64)
	V589V	2	0	0	0	0	2 (1.28)
	V650F	1	0	0	0	0	1 (0.64)
	A676S	1	0	0	0	0	1 (0.64)

*Pfcrt* (*n* = 153)	M74I^*a*^	18	19	6	10	0	53 (34.64)
	N75E^*b*^	18	19	6	10	0	53 (34.64)
	K76T^*c*^	18	19	6	10	0	53 (34.64)

*Pfmdr1* (*n* = 158)	N86Y	10	2	2	1	0	15 (9.49)
	G102G	0	0	0	1	0	1 (0.63)
	I107V	0	1	0	0	0	1 (0.63)
	Y184F	19	19	19	19	4	80 (50.63)

*Pfdhfr* (*n* = 145)	N51I	38	39	26	28	7	138 (95.17)
	C59R	38	38	25	29	7	137 (94.48)
	S108N	39	39	28	31	7	144 (99.31)
	D139V	0	0	0	0	0	0 (0)
	I164L	0	0	0	0	0	0 (0)

*Pfdhps* (*n* = 152)	I431V	11	13	1	6	2	33 (21.71)
	S436A	19	18	9	16	4	66 (43.42)
	S436Y	1	0	0	0	0	1 (0.66)
	A437G	40	36	26	28	6	136 (89.47)
	K540E	1	4	5	0	1	11 (7.24)
	A581G	7	11	2	6	2	28 (18.42)
	A613S	15	12	6	12	2	47 (30.92)

a Mixed mutation M74M/I included.

b Mixed mutation N75N/E/D/K included.

c Mixed mutation K76K/T included.

### Prevalence and patterns of *Pfcrt* mutations.

Of the 165 P. falciparum isolates from Nigeria described above, 153 were sequenced successfully. Wild-type (CVMNK), mutant-type (CVIET), and mixed mutant-type (CVM/IN/E/D/KK/T) *Pfcrt* alleles were detected (Tables 1 and 2). Among these alleles, the wild-type CVMNK allele was the most prevalent (65.36%; 100/153), followed by the triple mutant CVIET allele (29.41%; 45/153). Only eight (5.23%) isolates carried the mixed mutant type. By year of collection, the percentages of the triple mutation (CVIET) were 33.33% (17/51) in 2016, 39.02% (16/41) in 2017, 14.29% (4/28) in 2018, and 27.50% (19/29) in 2019. Regarding the mixed mutation (CVM/IN/E/D/KK/T), the proportions in 2017, 2018, and 2019 were similar, at about 7%, while the proportion was lower (1.96%; 1/51) in 2016. No mutant type was observed in 2020 due to the limited sample size.

**TABLE 2:** Prevalence of haplotypes of *Pfcrt*, *Pfmdr1*, *Pfdhfr*, and *Pfdhps* in isolates of P. falciparum imported to Zhejiang Province from Nigeria

Gene	Haplotype	Codon^*a*^	No. of isolates (%)					
			2016	2017	2018	2019	2020	Total
*Pfcrt* (*n* = 153)	Wild type	C_72_V_73_M_74_N_75_K_76_	33 (67.41)	22 (53.66)	22 (78.57)	19 (65.52)	4 (100.00)	100 (65.36)
	Triple mutant	CVIET	17 (33.33)	16 (39.02)	4 (14.29)	8 (27.50)	0	45 (29.41)
	Mixed mutant	CVM/I N/E/D/K K/T	1 (1.96)	3 (7.32)	2 (7.14)	2 (6.90)	0	8 (5.23)

*Pfmdr1* (*n* = 158)	Wild type	N_86_I_107_Y_184_	25 (50.00)	19 (46.34)	10 (34.48)	12 (38.71)	3 (42.86)	69 (43.67)
	Single mutant	YIY	6 (12.00)	2 (4.88)	0	0	0	8 (5.06)
		NVY	0	1 (2.44)	0	0	0	1 (0.63)
		NIF	15 (30.00)	19 (46.34)	17 (58.62)	18 (58.06)	4 (57.14)	73 (46.20)
	Double mutant	YIF	4 (8.00)	0	2 (6.90)	1 (3.23)	7 (50.00)	7 (4.43)

*Pfdhfr* (*n* = 145)	Wild type	N_51_C_59_S_108_D_139_I_164_	1 (2.50)	0	0	0	0	1 (0.69)
	Single mutant	NCNDI	0	0	1 (3.57)	2 (6.45)	0	3 (2.07)
	Double mutant	ICNDI	1 (2.50)	1 (2.56)	2 (7.14)	0	0	4 (2.76)
		NRNDI	1 (2.50)	0	1 (3.57)	1 (3.23)	0	3 (2.07)
	Triple mutant	IRNDI	37 (92.50)	38 (97.44)	24 (85.71)	28 (90.32)	7 (100.00)	134 (92.41)

*Pfdhps* (*n* = 152)	Wild type	I_431_S_436_A_437_K_540_A_581_A_613_	1 (2.22)	1 (2.50)	2 (6.90)	2 (6.45)	0	6 (3.95)
	Single mutant	IAAKAA	3 (6.67)	2 (5.00)	1 (3.45)	1 (3.23)	1 (14.29)	8 (5.26)
		ISGKAA	17 (37.78)	14 (35.00)	12 (41.38)	11 (35.48)	2 (28.57)	56 (36.84)
		ISAKAS	0	1 (2.50)	0	0	0	1 (0.66)
	Double mutant	IAGKAA	5 (11.11)	3 (7.50)	3 (10.34)	4 (12.90)	1 (14.29)	16 (10.53)
		ISGEAA	1 (2.22)	4 (10.00)	5 (17.24)	0	1 (14.29)	11 (7.24)
		ISGKAS	3 (6.67)	0	0	1 (3.23)	0	4 (2.63)
		VSGKAA	2 (4.44)	0	0	0	0	2 (1.32)
		IYAKAS	1 (2.22)	0	0	0	0	1 (0.66)
	Triple mutant	IAGKAS	3 (6.67)	1 (2.50)	4 (13.79)	5 (16.13)	0	13 (8.55)
		VAGKAA	0	2 (5.00)	0	0	0	2 (1.32)
		VSGKAS	1 (2.22)	0	0	0	0	1 (0.66)
		VSGKGA	0	2 (5.00)	0	0	0	2 (1.32)
		ISGKGS	0	0	1 (3.45)	1 (3.23)	0	2 (1.32)
	Quadruple mutant	IAGKGS	0	1 (2.50)	0	0	0	1 (0.66)
		VAGKGA	1 (2.22)	0	0	0	0	1 (0.66)
		VAGKAS	1 (2.22)	1 (2.50)	0	0	0	2 (1.32)
	Quintuple mutant	VAGKGS	6 (13.33)	8 (22.50)	1 (3.45)	5 (16.13)	2 (28.57)	22 (14.47)

a The underlined amino acid of the haplotypes indicated that it was mutant.

### Prevalence and patterns of *Pfmdr1* mutations.

A total of 158 samples were assessed successfully for the *Pfmdr1* codon polymorphisms N86F and Y184F (Tables 1 and 2). Point mutation N86Y was found in 9.49% (15/158) of the isolates, and Y184F was observed in half of the isolates (50.63%; 80/158). In addition, sequence alignments revealed that one sample from 2019 harbored a nucleotide mutation, T to C at position 306, and one sample from 2017 carried an amino acid mutation, isoleucine to valine at position 107. Analysis of the *Pfmdr1* haplotype prevalence showed that a single mutation type (NIF) was observed the most frequently (46.20%; 73/158), followed by the wild type (NIY) (43.67%, 69/158). A double mutant (YIF) was also found in 4.43% (7/158) of the samples.

### Prevalences and patterns of *Pfdhfr* mutations.

The haplotypes of *Pfdhfr* were determined for all 165 samples, but only 145 samples were detected successfully. Point mutations N51I, C59R, S108N, D139V, and I164L were recorded (Tables 1 and 2). Point mutation S108N predominated, at 99.31% (144/145). In addition, mutations N51I and C59R were frequently observed in 95.17% (138/145) and 94.48% (137/145) of the samples, respectively. However, there was no D139V or I164L mutation in these isolates. Regarding haplotypes of *Pfdhfr*, the prevalence of the triple mutation IRNDI was the highest (92.41%; 134/145). The proportions of the wild type, single mutations, and double mutations were as low as 0.69% to 2.76%.

### Prevalence and patterns of *Pfdhps* mutations.

Of the 165 parasite-positive samples, we successfully amplified the *Pfdhps* gene from 152 isolates. Nucleotide polymorphisms at positions 431, 436, 437, 540, 581, and 613 were identified. The results for *Pfdhps* mutations are shown in Tables 1 and 2. Seven single nucleotide polymorphisms were observed. The highest-frequency point mutation was A437G (89.47%; 136/152), which was followed by S436A (43.42%; 66/152) and A613S (30.92%; 47/152). Notably, we also found I431V in 21.71% of the samples. For the haplotype analysis, the diversity of the mutant type was recorded. Specifically, haplotype ISGKAA predominated, at 36.84% (56/152), and double mutant type IAGKAA and triple mutant type IAGKAS were found in 10.53% (16/152) and 8.55% (13/152) of the samples, respectively. It is important to note that the combination of A437G and K540E was found in 7.24% (11/152) of the samples. There was no obvious pattern between years.

### Patterns of *Pfdhfr*-*Pfdhps* mutations.

A total of 24 haplotypes were observed for *Pfdhfr*-*Pfdhps* mutations (Table 3). A quadruple mutant (IRNDI-ISGKAA) was the most frequent (48/142; 34.04%), followed by an octuple mutant (IRNDI-VAGKGS), at a frequency of 15.60% (22/141), and a quintuple mutant (IRNDI-IAGKAA), at a frequency of 11.35% (16/141). The quadruple combination of *Pfdhfr*-*Pfdhps* mutations (N51I, N59R, and S108N in *Pfdhfr* and A437G in *Pfdhps*) conferring partial resistance was found in 112 (79.43%) of the isolates. The quintuple combination (N51I, N59R, and S108N in *Pfdhfr* and A437G and K540E in *Pfdhps*) conferring full resistance was found in 8 (5.67%) isolates in 2016 (1 isolate), 2017 (2 isolates), 2018 (4 isolates), and 2020 (1 isolate). The sextuple combination (N51I, N59R, and S108N in *Pfdhfr* and A437G, K540E, and A581G in *Pfdhps*) conferring superresistance was not seen.

**TABLE 3:** Prevalence of *Pfdhps*-*Pfdhfr* haplotypes in P. falciparum isolates imported to Zhejiang Province from Nigeria

Haplotype	Codon^*a*^	No. of isolates (%)
Single mutant	NCNDI-ISAKAA	2 (1.42)

Double mutant	NRNDI-ISAKAA	1 (0.71)

Triple mutant	ICNDI-ISGKAA	3 (2.13)
	IRNDI-ISAKAA	3 (2.13)
	NRNDI-IAAKAA	1 (0.71)

Quadruple mutant	IRNDI-ISGKAA	48 (34.04)
	IRNDI-IAAKAA	7 (4.96)
	IRNDI-ISAKAS	1 (0.71)
	ICNDI-VSGKAA	1 (0.71)
	NCSDI-VAGKAS	1 (0.71)

Quintuple mutant	IRNDI-IAGKAA	16 (11.35)
	IRNDI-ISGEAA	8 (5.67)
	IRNDI-ISGKAS	4 (2.84)
	IRNDI-VSGKAA	1 (0.71)
	NRNDI-IAGKAS	1 (0.71)

Sextuple mutant	IRNDI-IAGKAS	10 (7.09)
	IRNDI-ISGKGS	2 (1.42)
	IRNDI-VAGKAA	3 (2.13)
	IRNDI-VSGKAS	1 (0.71)
	IRNDI-VSGKGA	2 (1.42)

Septuple mutant	IRNDI-VAGKAS	1 (0.71)
	IRNDI-VAGKGA	1 (0.71)
	IRNDI-IAGKGS	1 (0.71)

Octuple mutant	IRNDI-VAGKGS	22 (15.60)

Total		141 (100.00)

a The underlined amino acid of the haplotypes indicated that it was mutant.

## DISCUSSION

Due to the spread of drug-resistant parasites worldwide, especially the emergence of artemisinin-resistant P. falciparum *k13* R561H mutant parasites in Rwanda (8), it is becoming increasingly important to monitor updated antimalarial resistance in Africa. Therefore, in the present study, we collected P. falciparum isolates imported from Nigeria to Zhejiang Province, China, between 2016 and 2020 to provide updated insight into the *k13*, *Pfcrt*, *Pfmdr1*, *Pfdhfr*, and *Pfdhps* genetic profiles. It is estimated that about one-quarter of malaria patients (22.78% to 26.42% between 2016 and 2020) in Zhejiang returned from Nigeria. The majority of these patients were infected by P. falciparum. The number of P. falciparum cases declined from 53 in 2016 to 9 in 2020, which could be explained by a sharp decrease in migrant workers because of the influence of coronavirus disease 2019 (COVID-19).

ACT was introduced in 2005 as the first-line treatment for malaria in Nigeria (4, 5), and AL is most commonly used in ACT (12). Ten years following ACT deployment as a first-line antimalarial treatment in Nigeria, a significant decline in the early responses of childhood P. falciparum infections to AL and artesunate-amodiaquine was reported and raised concern. However, we observed no validated molecular markers in the *k13* propeller domain associated with artemisinin resistance, indicating an optimistic status concerning artemisinin resistance in Nigeria. Similarly, no validated *k13* polymorphism associated with artemisinin resistance was seen in P. falciparum isolates in previous reports from the Lagos, Osun, and Anambra States of Nigeria (12–14). Additionally, our data showed 3.21% nonsynonymous mutations, P441S, N458D, V650F, and A676S, in five isolates. The A676S substitution was previously discovered in Ghana and Southeast Asia, and therapeutic efficacy data from Ghana still support its sensitivity to AL (15–17). As no clinical study or *in vitro* tests were performed here, the nonsynonymous mutations P441S, N458D, and V650F could not be correlated with artemisinin sensitivity. Therefore, this observation warrants a larger sample size to monitor these molecular markers as well as their response to antimalarials to evaluate their potential influence on and correlation with drug resistance.

Mutations at positions 72 to 76 of *Pfcrt* are thought to play a decisive role in conferring resistance to CQ (18). In Nigeria, CQ-resistant strains of P. falciparum were first observed in the southern region in 1987 and then spread across the country (4). ACT combined with artemether-lumefantrine was introduced as a first-line antimalarial treatment in 2005 (3, 4). Eleven years after the withdrawal of CQ, our study showed a low frequency of mutant type CVIET in Nigeria. Specifically, the overall prevalence of *Pfcrt* M74I, N75E, and K76T in the present study was found to be 34.64%, and 65.36% of the isolates carried the wild-type (CVMNK) *Pfcrt* gene, followed by triple mutants (29.41%), which is similar to the prevalences reported previously in isolates from Nigeria and other African countries such as Angola, the Republic of Guinea, Ghana, Equatorial Guinea, Cameroon, and Chad (19, 20). The high proportion of wild-type *Pfcrt* codons 72 to 76 indicates a return of CQ-sensitive P. falciparum isolates to Nigeria. There were also high levels of CQ-sensitive haplotypes of P. falciparum in China (21), Tanzania (22), Malawi (23), and Kenya (24) after CQ was no longer on the first-line list for the treatment of P. falciparum, which reflected decreasing drug pressure on the parasite population. However, our results are in contrast to those of several previous studies. For instance, the prevalence of haplotype CVIET was 76.37% in asymptomatic infections in Anambras, southeastern Nigeria (4); similarly, another two investigations carried out in Lagos and Edo in southwestern Nigeria showed that 38.7% and 4.2% of the isolates were of the wild type, respectively (25, 26). The high proportion of CVIET in regions of Nigeria might be explained by prescription practices for CQ in private facilities that do not follow the national drug policy (3). Conclusions cannot yet be drawn nationally in Nigeria on the dominant *Pfcrt* codons 72 to 76. It is necessary to perform systematic investigations in Nigeria to reevaluate the national *Pfcrt* situation and CQ efficacy.

*Pfmdr1* is regarded as a pivotal factor in antimalarial drug resistance (10). We detected 9.49% of isolates with the N86F mutation in *Pfmdr1*. This finding was in line with the results of previous studies by Ikegbunam et al. in southeastern Nigeria (8.54%) (4) and Adam et al. in Kano State (11.3%) (27). Furthermore, our study revealed a higher proportion (50.63%) of isolates with the Y184F mutation, while a prevalence of only 29.27% was observed by Ikegbunam et al. The frequencies of *Pfmdr1* gene mutations varied among the isolates in previous reports. Dokunmu et al. observed that 24% of isolates carried the N86F mutation among asymptomatic and symptomatic infections in Ota, southwestern Nigeria (28). In Lagos and Ede, Osun State, the N86F mutation was present in 44% of the isolates sequenced, while the Y184F mutation was found at a higher prevalence of 86.75% (13). The *Pfmdr1* N86F mutation has been linked to lower parasite responses to a broad range of antimalarials, including CQ, artemisinin derivatives, mefloquine, and lumefantrine, while the Y184F variant has a limited effect. However, their combination (N86F and Y184F double mutant) is known to be associated with reduced piperaquine susceptibility (10). Fortunately, the frequency of the *Pfmdr1* double mutant type (N86Y and Y184F) in the present study was as low as 4.73% (7/158). However, routine surveillance is still recommended to monitor the temporal and spatial distribution of molecular markers of *Pfmdr1* given their varying regional prevalence in Nigeria (27).

Our study showed high frequencies of P. falciparum isolates with *Pfdhfr* and *Pfdhps* mutations in circulation in Nigeria. Point mutations N51I, C59R, and S108N in *Pfdhfr* were prevalent, which is comparable to the results of some other previously reported studies of asymptomatic pregnant women from Calabar as well as patients and asymptomatic persons from Lagos State, Nigeria (29, 30). We detected the newly discovered mutant I431V in 21.71% of isolates between 2016 and 2020, which was comparable to or higher than the rates reported in previous studies from Nigeria (7, 29, 31). Although no conclusive evidence was obtained about the effect of I431V on the drug response, molecular surveillance supports that I431V has been common in Nigeria and spread to neighboring countries (31, 32). In particular, we found an octuple mutant type, VAGKGS, in 22 isolates, for a prevalence of 15.60%, highlighting the spread of highly mutated *Pfdhps* in Nigeria. The significance of I431V remains unknown; however, based on our findings, it has become crucial to evaluate the impact of the combination of I431V with A437G, K540E, and A581G in *Pfdhps*. The high prevalence (92.41%) of triple mutant haplotype IRNDI of *Pfdhfr* found in this study confirms previously reported research showing that the efficacy of SP may be threatened in Nigeria (29, 30). Our analysis showed that the single mutant haplotype ISGKAA dominated in Nigeria. This was consistent with data from previous reports in Nigeria as well as other African countries (33). Alarmingly, 7.24% of the samples in our survey harbored a *Pfdhps* double mutation at codons 437 and 540, which is known for its contribution to clinical resistance to SP when acting in combination with triple mutant *Pfdhfr* (N51I, N51I, and S108N) (34). Considering *Pfdhfr* and *Pfdhps* together, a fully resistant set of quintuple *Pfdhfr-Pfdhps* mutations, A437G and K540E in *Pfdhps* and N51I, N59R, and S108N in *Pfdhfr*, has rarely been described in West Africa, whereas the combination of A437G and triple mutant *Pfdhfr*, conferring partial resistance, is common (35). To be specific to Nigeria, the quintuple *Pfdhfr-Pfdhps* mutation was not detected in recent studies (29, 30, 36). However, we found it in 8 (5.67%) isolates, along with the quadruple *Pfdhfr-Pfdhps* mutation in 112 (79.43%) isolates, indicating potentially increased resistance to SP in Nigeria. The current study clearly reveals the emergence and spread of the highly prevalent quintuple *Pfdhfr-Pfdhps* mutant in Nigeria, highlighting the importance of the continuous and strategic surveillance of molecular markers and the efficacy of SP.

The current study was limited by the region-specific distribution of molecular markers because data on the region of origin of imported cases were not collected when the epidemiological investigation was conducted, according to national guidelines. Second, the small sample size limited further interpretation by year. However, our analysis still provides data on molecular markers associated with resistance to artemisinin, CQ, and SP, from the view of P. falciparum isolates imported from Nigeria.

In conclusion, we demonstrated the prevalences and patterns of mutations in the *k13*, *Pfcrt*, *Pfmdr1*, *Pfdhfr*, and *Pfdhps* genes in P. falciparum isolates imported from Nigeria to Zhejiang Province, China, between 2016 and 2020. Limited polymorphism in the *k13* gene was detected. The wild-type CVMNK haplotype of *Pfcrt* became predominant. A low frequency of the double mutant type (N86Y and Y184F) in *Pfmdr1* was observed. The prevalences of the triple mutation (IRNDI) in *Pfdhfr* and the single mutation (SGKAA) in *Pfdhps* were the highest. We found a quintuple combination of *Pfdhfr-Pfdhps* mutations in eight samples, which was rarely described in Nigeria, indicating the continued emergence and spread of SP resistance. The current study demonstrated a high frequency of wild-type *Pfcrt*, limited polymorphism of *Pfmdr1*, and a high prevalence of *Pfdhfr* and *Pfdhps* mutants. In this work, we present a comprehensive set of molecular markers for drug resistance from a sample of imported cases. Based on our findings, it has become crucial to evaluate the impact of the emerging “fully resistant” type of *Pfdhfr*-*Pfdhps* as well as its combination with I431V on the efficacy of SP in Nigeria.

## MATERIALS AND METHODS

### Study site.

Zhejiang Province is located in eastern China. The land area of Zhejiang is 105,500 km^2^, and the local population size was 50.38 million in 2019. It is located in the center of the subtropical zone, with an average annual temperature of 15°C to 18°C and annual rainfall of 1,200 to 2,000 mm. Anopheles sinensis is reported to be the only species of mosquito to transmit malaria. Labor export and trading are common between Zhejiang and Nigeria, and many migrant workers and business people travel between the two places each year.

### Sample collection and DNA extraction.

We investigated 165 P. falciparum infections imported from Nigeria to Zhejiang Province between January 2016 and December 2020. Individuals with malaria-related symptoms and positive results by microscopy or rapid diagnostic tests (RDTs) are reported by hospitals or clinics according to national guidelines on malaria diagnosis. The local Center for Disease Control and Prevention (CDC) carried out an epidemiological investigation for each patient, including a detailed travel history, to track parasite origins. Thick and thin blood smears and PCR were used by the Zhejiang Provincial CDC to double-check and confirm the species. Whole blood was collected from each case before antimalarial treatment. All blood samples were stored at −80°C until use. Genomic DNA was extracted using the QIAamp DNA minikit (Qiagen Inc., Hilden, Germany).

### DNA amplification and sequencing.

Multiple molecular markers were detected, including the *k13*, *Pfcrt*, *Pfmdr1*, *Pfdhfr*, and *Pfdhps* genes. *k13*, *Pfmdr1*, *Pfdhfr*, and *Pfdhps* were detected by nested PCR, and *Pfcrt* was detected by regular PCR, as previously described (37–40). Sequences for positions 72 to 76 of *Pfcrt* and *Pfmdr1* haplotypes (codons 86 and 184), *Pfdhfr* haplotypes (codons 51, 59, 108, 139, and 164), and *Pfdhps* haplotypes (codons 431, 436, 437, 540, 581, and 613) were amplified. The primers used for PCR are listed in Table S1 in the supplemental material (37, 38). Proofreading polymerase was used for each reaction to prevent amplification errors, which was contained in the high-fidelity PCR master mix (Sangon Biotech Co. Ltd., Shanghai, China). The cycling conditions for the *k13*, *Pfcrt*, *Pfdhfr*, and *Pfdhps* genes were described in previous studies (39, 40). Amplification conditions for both PCR rounds for *Pfmdr1* were as follows: 1 cycle at 95°C for 5 min; 30 cycles at 95°C for 1 min, 54°C for 90 s, and 72°C for 1 min; and 1 extension cycle at 72°C for 10 min. PCR products were sequenced by Sangon Biotech.

### Data availability.

Mega version 7.0.26 (https://www.megasoftware.net/) was used to align amplicon sequences to reference sequences retrieved from the NCBI database. The GenBank accession numbers for reference sequences are XM_001350122.1 for *k13*, NC_004328.3 for *Pfcrt*, X56851.1 for *Pfmdr1*, NC_004318.2 for *Pfdhfr*, and XM_001349382.1 for *Pfdhps*. A database was constructed using Microsoft Excel 2017.
