# Supplementary material for: Molecular Epidemiology of Drug Resistance Genes in Plasmodium falciparum Isolates Imported from Nigeria between 2016 and 2020: Continued Emergence of Fully Resistant Pfdhfr-Pfdhps Alleles
Source: Microbiol Spectr. 2022 Sep 15;10(5):e00528-22. doi: 10.1128/spectrum.00528-22 (PMC9604097; doi:10.1128/spectrum.00528-22)
Supplement: Supplemental file 1 — Table S1, Table S2. Download spectrum.00528-22-s0001.pdf, PDF file, 0.1 MB [file spectrum.00528-22-s0001.pdf]

TABLE S1 Primers for *k13*, *Pfcrt*, *Pfmdr1, Pfdhfr and Pfdhps* propeller genotyping assay

Gene	Round	Primer name	Sequence (5'→3')
k13	Primary	K13-1-F	CGGAGTGACCAAATCTGGGA
		K13-1-R	GGGAATCTGGTGGTAACAGC
	Secondary	K13-2-F	GCCAAGCTGCCATTCATTTG
		K13-2-R	GCCTTGTTGAAAGAAGCAGA
Pfcrt	Primary	*Pfcrt*-1-F	CCCTTGTCGACCTTAACAGATG
		Pfcrt-1-R	AAAATGACTGAACAGGCATCTAAC
	Secondary	*Pfcrt*-2-F	TCTTGGTAAATGTGCTCATGTG
		Pfcrt-2-R	AAAGTTGTGAGTTTCGGATGTT
Pfmdr1	Primary	*Pfmdr1*-1-F	TTAAATGTTTACCTGCACAACATAGAAAATT
		Pfmdr1-1-R	CTCCACAATAACTTGCAACAGTTCTTA
	Secondary	*Pfmdr1*-2-F	TGTATGTGCTGTATTATCAGGA
		Pfmdr1-2-R	CTCTTCTATAATGGACATGGTA
Pfdhfr	Primary	*Pfdhfr*-1-F	TTTATGATGGAACAAGTCTGC
		Pfdhfr-1-R	CTAGTATATACATCGCTAACA
	Secondary	*Pfdhfr*-2-F	TGATGGAACAAGTCTGCGACGTT
		Pfdhfr-2-R	CTGGAAAAAATACATCACATTCATATG
Pfdhps	Primary	*Pfdhps*-1-F	GATTCTTTTTCAGATGGAGG
		Pfdhps-1-R	TTCCTCATGTAATTCATCTGA
	Secondary	*Pfdhps*-2-F	AACCTAAACGTGCTGTTCAA
		Pfdhps-2-R	AATTGTGTGATTTGTCCACAA

TABLE S2 Temporal distribution of imported *P.falciparum* cases from Nigeria in Zhejiang province between 2016 and 2020

Year	Total imported malaria cases in Zhejiang	Malaria cases from Nigeria (%)[a]	*P.falciparum* cases from Nigeria (%)[b]
2016	246	59 (23.98)	53 (89.83)
2017	204	48 (23.53)	42 (87.50)
2018	158	36 (22.78)	29 (80.56)
2019	178	45 (25.28)	33 (73.33)
2020	53	14 (26.42)	8 (57.14)
Total	839	202 (24.08)	165 (81.68)

a, percent of malaria cases from Nigeria out of total cases in Zhejiang.

b, percent of *P.falciparum* cases out of total malaria cases from Nigeria
